# The morphological regeneration and functional restoration of bladder defects by a novel scaffold and adipose-derived stem cells in a rat augmentation model

**DOI:** 10.1186/s13287-017-0597-z

**Published:** 2017-06-24

**Authors:** Qiong Wang, Dong-dong Xiao, Hao Yan, Yang Zhao, Shi Fu, Juan Zhou, Zhong Wang, Zhe Zhou, Ming Zhang, Mu-Jun Lu

**Affiliations:** 1 Department of Urology and Andrology, Shanghai Renji Hospital, Shanghai Jiao Tong University, School of Medicine, No. 145 Middle Shandong Road, Shanghai, 200001 People’s Republic of China; 2 Department of Urology, Shanghai Ninth People’s Hospital, School of Medicine, Shanghai Jiao Tong University, Shanghai, 200011 People’s Republic of China

**Keywords:** Adipose-derived stem cells, Bladder regeneration, Functional restoration, Scaffold

## Abstract

**Background:**

Due to the multilineage differentiation ability and paracrine role of adipose-derived stem cells (ASCs) for bladder defect repair, various scaffolds have been applied in combination with ASCs to promote bladder regeneration and restore bladder function. However, the low survival rate of ASCs and the difficulty of promoting bladder functional recovery are still unsolved. To explore these problems, we investigated the feasibility of a novel scaffold seeded with ASCs in a rat model of bladder augmentation.

**Methods:**

A novel autologous myofibroblast (AM)-silk fibroin (SF) scaffold was harvested after subcutaneously prefabricating the bladder acellular matrix grafts (BAMG) and SF by removing the BAMG. The AM-SF scaffolds were then seeded with ASCs (AM-SF-ASCs). Fifty percent supratrigonal cystectomies were performed followed by augmenting the cystectomized defects with AM-SF scaffolds or AM-SF-ASCs. The histological and functional assessments of bladders were performed 2, 4, and 12 weeks after surgery while the ASCs were tracked in vivo.

**Results:**

For bladder tissue regeneration, immunofluorescence analysis revealed that AM-SF-ASCs (the experimental group) promoted better morphological regeneration of the urothelium, vessels, bladder smooth muscle, and nerve than AM-SF scaffolds (the control group). Regarding functional restoration, the AM-SF-ASC group exhibited higher bladder compliance and relatively normal micturition pattern compared to the AM-SF group. In addition, a certain number of surviving ASCs could be found in vivo 12 weeks after implantation, and some of them had differentiated into smooth muscle cells.

**Conclusions:**

The AM-SF scaffolds with ASCs could rapidly promote bladder morphological regeneration and improved bladder urinary function. In addition, the bag-shaped structure of the AM-SF scaffold can improve the survival of ASCs for at least 12 weeks. This strategy of AM-SF-ASCs has a potential to repair large-scale bladder defects in the clinic in the future.

## Background

Critical defects in the bladder caused by tumor resection, tuberculosis, and other diseases are a major clinical issue which are traditionally treated by enterocystoplasty [1]. However, enterocystoplasty not only causes lesions to the intestinal tract but also leads to possible complications. A variety of synthetic polymers and biological materials have been applied to facilitate bladder defect repair [2–4].

Silk fibroin (SF) is a natural derivative of silkworm cocoons that possesses tuneable mechanical properties, superficial biodegradability, and plasticity [4]. However, it was reported that simple SF structures may increase the risk of urinary stones and urinary leakage [5]. Myofibroblasts, a fibroblast-like cell containing actin, are involved in contraction and fibrosis of wound healing by secreting extracellular matrix and cell contractions [6–8]. It has been shown that autologous myofibroblasts (AMs) have superior potential for facilitating the repair of hollow smooth muscle organs, such as the uterus, arteries, the vas deferens, the bladder, and urethra [9, 10]. Adipose-derived stem cells (ASCs) are pluripotent stem cells that are advantageous for isolating, harvesting, and expanding [3]. Studies have shown that ASCs can differentiate into multiple mature cell types, such as urothelium, osteocytes, and lipocytes [11, 12]. Other studies have indicated that ASCs can secrete various growth factors including angiopoietin-1 (Ang-1), vascular endothelial growth factor (VEGF), nerve growth factor (NGF), brain-derived neurotropic factor (BDNF), and glial cell-derived neurotropic factor (GDNF) which could promote angiogenesis and nerve axon growth in vitro [13]. Therefore, ASCs have been widely used in tissue engineering for bladder regeneration [3, 14, 15]. However, the low survival rate of ASCs limits their further applications. Moreover, the specific mechanism by which ASCs promote bladder regeneration remains unclear [16].

In this study, we investigated the feasibility of AM-SF scaffolds seeded with ASCs in facilitating bladder augmentation and their roles for bladder morphological regeneration and functional restoration.

## Methods

### Scaffold preparation

Bladder acellular matrix grafts (BAMG)-SF scaffold was synthesized as previously described [17]. Briefly, bladder tissues were harvested from 3-month-old pigs and rinsed with phosphate-buffered saline (PBS). The fatty and collagenous connective tissues around the urinary bladder were removed using scissors, and then the urothelium, muscle, and serosal layers were mechanically removed. Afterwards, the submucosal layer of the bladder was washed with double distilled water for 2 days at 4 °C and soaked in 0.2% Triton X-100 (Sigma, St. Louis, MO, USA) and 0.1% (w/v) ammonium hydroxide for 7 days at room temperature. The solution was refreshed every 2 days. After cutting into 15 mm × 15 mm squares, the resulting BAMG was placed in a mold, and 300 μl SF solution (2% w/v) was directly poured onto the rough layer of the BAMG. The BAMG-SF was harvested after freezing and lyophilizing. The grafts were sterilized with ethylene oxide before application. The decellularization efficacy and mechanical testing of the BAMG-SF was evaluated in our previous study [17]. The BAMG-SF was cut into 1 cm × 1 cm squares, subcutaneously incubated in the backs of 8-week-old female Sprague-Dawley (SD) rats, and then harvested at 1, 3, 7, and 14 days after implantation for histological evaluation. The AM-SF scaffolds were harvested by removing the BAMG at 7 days after subcutaneous prefabrication.

### Histological and immunological examination

At 1, 3, 7, and 14 days after subcutaneous prefabrication of the BAMG-SF, the graft and tissues around it were harvested and immediately fixed in 4% formaldehyde for 4 h followed by dehydration through a series of graded ethanol solutions and embedded in paraffin. Paraffin-embedded tissue was sectioned onto glass slides, and the slides were deparaffinized at 60 °C for 30 min followed by treatment with xylenes, graded ethanol, and double distilled water according to well-established protocols [18]. Hematoxylin and eosin (HE) staining, Masson’s Trichrome staining, and immunohistochemistry staining for myeloperoxidase (MPO) and CD68 markers were performed to examine the incubated BAMG-SF.

The AM-SF was harvested as described above and HE, Masson’s Trichrome staining, and immunofluorescence staining for vimentin, α-smooth muscle actin (α-SMA) were performed to examine the composition of AM-SF.

### Scanning electron microscopy

Scanning electron microscopy (SEM) was used to probe the surface morphology, thickness, and space of the AM-SF scaffolds. AM-SF was fixed in 2.5% glutaraldehyde for 4 h and lyophilized for 2 days, then sputter-coated (Balzers Union 07120/135, Germany) with 10 nm platinum/gold. Images were recorded using a JEOL 6360 LV microscope (Tokyo, Japan) at different magnifications and with different views.

### ASC culture, identification, and labeling

The adipose tissues were isolated from the groin area of SD rats and rinsed three times with 0.25% chloromycetin and PBS. They were cut into pieces and digested with 0.1% type IV collagenase (Sigma-Aldrich) under continuous oscillation at 37 °C for 1 h. Then, the solution was centrifuged at 1500 rpm for 5 min at 37 °C. The supernatant was removed, and the precipitate was resuspended in Dulbecco’s modified Eagle’s medium (DMEM; Gibco/Invitrogen Corporation) containing 10% fetal bovine serum (FBS; Gibco/Invitrogen Corporation), and 1% penicillin-streptomycin solution (Gibco/Invitrogen Corporation), and the suspension was then filtered through a 200-μm nylon filter mesh to obtain a single cell suspension. The isolated ASCs were seeded in 10-cm cell culture plates and cultured at 37 °C with 5% humidified carbon dioxide. The culture medium was changed every 2 days and the cells were passaged when they had reached 80–90% confluence.

The identification of ASCs was carried out as in our previous study to assess differentiation into adipocytes and osteoblasts [3]. To track ASCs after transplantation in vivo, passage 3 ASCs were labelled with Cell Tracker CM-Dil (Invitrogen) as previously described [3]. In brief, ASCs were incubated with 1.5 μM CM-Dil at 37 °C for 5 min and then at 4 °C for 15 min, washed with PBS, and resuspended in DMEM containing 10% FBS before animal experiments.

In addition, passage 3 ASCs were seeded on slides for immunofluorescence evaluation of cytokeratin (CK), SM22α, NeuN, and CD31 to determine whether ASCs react with these antibodies.

### Experimental animals

Forty-six 8-week-old female SD rats were used in this study. Four rats were used to investigate several time points of subcutaneous implantation and find an optimal time point for subcutaneous prefabrication. The remaining 42 rats were divided into three groups: simple application of AM-SF scaffolds (control group, *n* = 18), AM-SF scaffolds seeded with ASCs (experimental group, *n* = 18), and simple cystotomy (sham operation group, *n* = 6). At 2, 4, and 12 weeks after bladder augmentation, the bladders of the 42 rats were harvested for evaluation.

### Rat bladder augmentation surgical technique

Rats were anesthetized by isoflurane inhalation and shaved to expose the skin of the middle back and lower abdomen. First, the 1 cm × 1 cm BAMG-SF-AM scaffold was harvested from the back after 1 week of incubation, and the fatty and collagenous connective tissues were removed. Second, a 1-cm incision was made in one side of the BAMG-SF-AM using a scalpel, and the BAMG was gently removed using forceps to leave the AM-SF scaffold. Third, the AM-SF scaffold was seeded with PBS (40 μL) or ASCs (40 μL, 10 × 10^7^ cells/mL) in the incision. Finally, the incision of the AM-SF-ASC scaffold was anastomosed using 8–0 polyglactin sutures (Johnson & Johnson Ltd) via the lock-stitch suture method. The incision in the back of the rat was sutured, and the rat was turned over to expose its abdomen. Then, a 1.5-cm lower abdominal midline incision was made, followed by dissection of the subcutaneous tissue, the rectal muscle, and the peritoneum to expose the bladder. A 50% supratrigonal cystectomy was performed from anterior to posterior followed by augmenting the cystectomized defect with an AM-SF scaffold or AM-SF-ASCs using 8–0 polyglactin sutures. Finally, a watertight seal was confirmed by filling the bladder with sterile saline via the lower urethra using a venous indwelling needle.

### Cystography

Cystography was performed in three groups at 2, 4, and 12 weeks after augmentation. After general anesthesia, 1 mL iopamidol (350 mg/mL; GE Healthcare) was injected into the bladder through a venous indwelling needle in the lower urethra. The X-ray film was obtained for each experimental subject.

### Histological examination

At 2, 4, and 12 weeks after augmentation, rats were euthanized by CO_2_ asphyxiation, and their bladders were excised for histological processing, as described above. The slides were stained with HE staining to observe general bladder reconstruction. CK AE1/AE3 (diluted 1:200, boiled; Abcam), SM22α (diluted 1:150, Zymed; Abcam), NeuN (diluted 1:150, boiled; Abcam) and CD31 (1:400 dilution, Zymed; Epitomics) were used to assess the regeneration of urothelium, smooth muscle cells, neurons, and blood vessels, respectively, via immunofluorescence according to standard procedures. Images were acquired for statistical analysis using a Nikon Eclipse TE2000-U fluorescence microscope (Nikon Instruments Inc, Melville, NY, USA).

### Urodynamics examination

Urodynamic parameters were measured 12 weeks after surgery, as previously described [19]. One end of the PE-50 tubing was exposed to a flame to form a funnel to prevent the tube from falling off the bladder. Then, a lower abdominal incision was made to expose the bladder, as described above. A tunnel serving as passage for the PE-50 tube was created from the back to the lower abdomen after a 1-cm incision was made on the dorsum. Next, a hole in the dome of the bladder was made to insert the PE-50 tube, which was fastened by 5–0 polyglactin sutures with a purse string suture. Finally, normal saline at room temperature was injected into the bladder by opening the PE-50 tube on the back to test urine leakage. The end of the PE-50 tube at the dorsum was connected via a T-shaped tube to a pressure transducer and a peristaltic pump. The bladder pressure and voiding volume were recorded during continuous infusion of room temperature saline (200 μL/min). The threshold micturition pressure (ΔP), maximal bladder capacity (ΔV), and bladder compliance (ΔV/ΔP) were recorded and evaluated.

### Composition analysis of bladder calculus

An infrared spectrometer was used to analyze stone sample composition at Renji Hospital, Shanghai Jiao Tong University School of Medicine, based on the position, intensity and shape of waveforms in the spectra. First, stone samples were rinsed with double distilled water and then dried at room temperature. Second, the samples were rubbed into powder and mixed with potassium bromide (KBr) at a ratio of 1:50. Finally, the samples were pressed into thin slices (thickness of 1 mm), and their analyzed spectra were analyzed using an infrared spectrum automatic analysis instrument (LIIR type).

### Statistical analysis

All quantitative data were evaluated using GraphPad Prism v5.0 Software. Statistical differences between the groups were analyzed by one-way analysis of variance (ANOVA) followed by Student’s *t* tests, and *p* < 0.05 was considered statistically significant. All data are expressed as the means ± standard deviations.

## Results

### Evaluation of BAMG-SF and AM-SF scaffolds

**Fig. 1:**
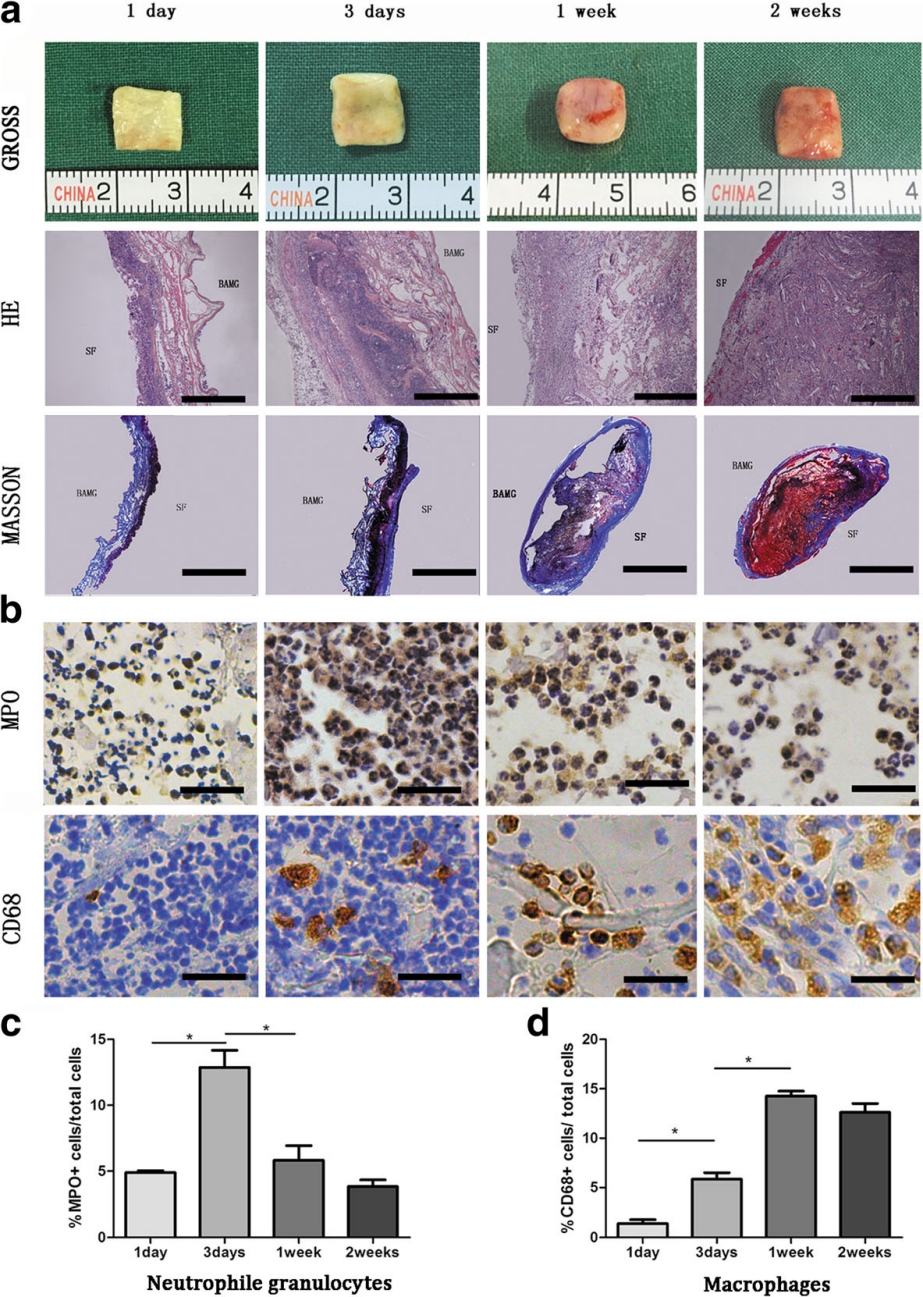
Histological and immunological observations of subcutaneously prefabricated bladder acellular matrix grafts-silk fibroin (*BAMG-SF*). **a** Gross morphology (*top row*) of BAMG-SF subcutaneously prefabricated for 1 day, 3 days, 1 week, and 2 weeks. *Scale bars* = 1 cm. Photomicrographs of BAMG-SF (longitudinal section of hematoxylin and eosin (*HE*; *middle row*; 50×, *scale bars* = 500 μm) and Masson staining (*bottom row*; 12.5×, *scale bars* = 2 mm). **b** Myeloperoxidase (*MPO*) staining (top row) for neutrophil granulocytes (acute inflammatory response; 400×, *scale bars* = 50 μm) and CD68 staining (*bottom row*) for macrophages (chronic inflammatory response; 400×, *scale bars* = 50 μm). Statistical analysis of **c** neutrophil granulocytes and **d** macrophages. *p < 0.05, between groups

**Fig. 2:**
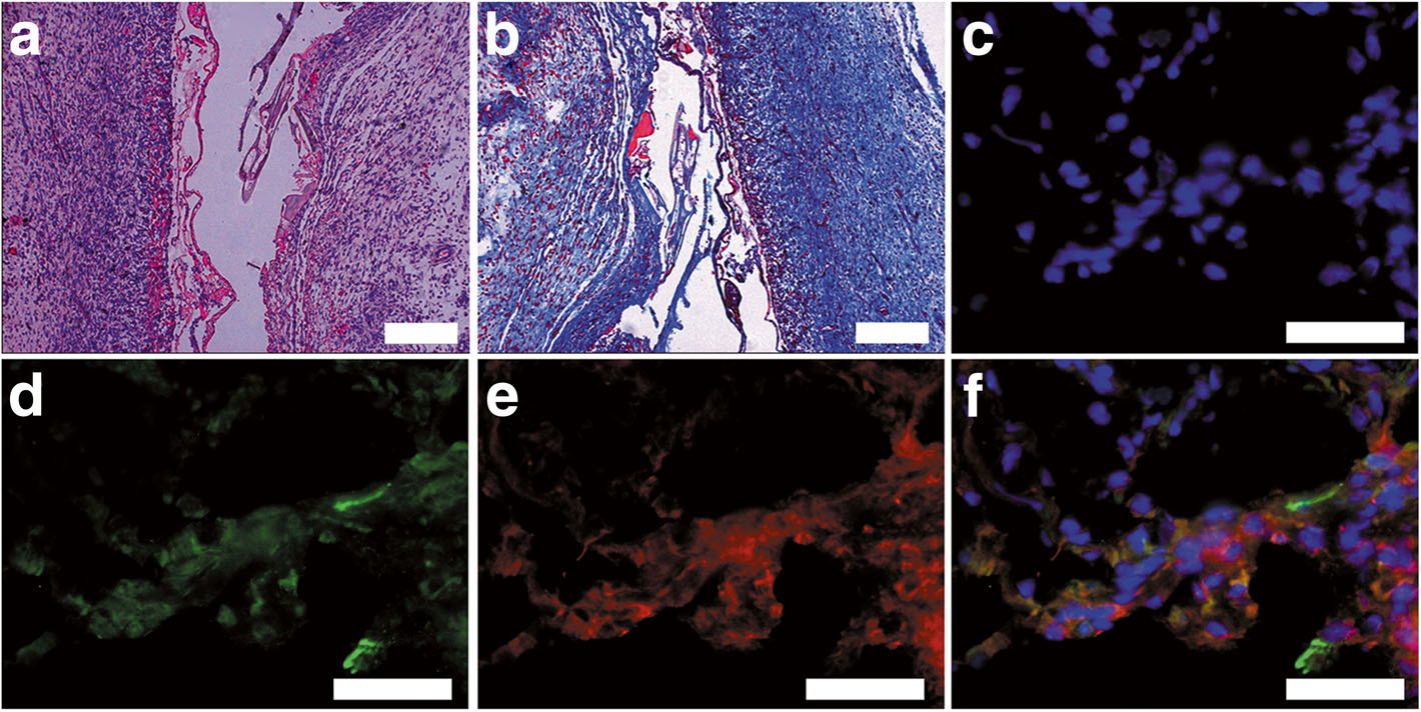
Histological and immunofluorescence observations of the AM-SF scaffolds. **a**,**b** HE and Masson staining; 100×, *scale bars* = 200 μm. **c** Photomicrograph of nuclei stained with DAPI; 400×, *scale bar* = 50 μm. **d**,**e** Photomicrograph of α-SMA (*green*) and vimentin (*red*) immunofluorescence; 400×, *scale bars* = 50 μm. **f** Merged images; 400×, *scale bar* = 50 μm

With the gradual increase in implantation time, the BAMG-SF was surrounded by the subcutaneous connective tissue (Fig. 1a). One day after subcutaneous implantation of the BAMG-SF, the SF surface was heavily invaded by MPO+ neutrophil granulocytes (acute inflammatory response) (Fig. 1b and c). Few CD68+ macrophages (chronic inflammatory response) were found in the scaffolds (1.39 ± 0.80%). In the 3-day group, the intrusion of neutrophil granulocytes was significantly increased compared to the 1-day group (12.86 ± 2.63% versus 4.89 ± 0.28%, *p* < 0.05; Fig. 1c), and the SF surface was surrounded by a layer of blue collagen fibers (Fig. 1a) that were determined to be myofibroblasts (Fig. 2). In addition, macrophages began to invade the graft. In the 1-week group, the intrusion of neutrophil granulocytes was significantly decreased compared with the 3-day group (5.81 ± 2.23% versus 12.86 ± 2.63%﻿, *p* < 0.05), and more macrophages invaded the BAMG-SF (14.25 ± 1.01% versus 5.84 ± 1.32%, *p* < 0.05). Two weeks after BAMG-SF implantation, the intrusion of neutrophil granulocytes and macrophages were not significantly different compared to the 1-week group (*p* > 0.05). The immunofluorescence double staining showed that the AM were simultaneously positive for α-SMA and vimentin, which presented markers of myofibroblasts (Fig. 2d and e).

### Scanning electron microscopy observations

**Fig. 3:**
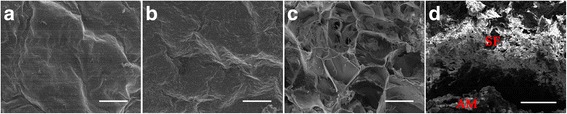
Photomicrographs of representative scanning electron microscopy images demonstrating AM-SF scaffold configurations. **a** The outward appearance of autologous myofibroblasts (*AMs*); *scale bar* = 100 μm. The inner appearance of **b** AMs and **c** silk fibroin (*SF*); *scale bars* = 100 μm. **d** The cross-sectional appearance; *scale bar* = 500 μm

The outward appearance of AMs directly contacting the abdominal cavity or bladder lumen was smooth (Fig. 3a). The inner appearance of the AM-SF scaffold contacting the ASCs consisted of two sides: the AM side (Fig. 3b) and the SF side (Fig. 3c). The SF side resembled a foam configuration with large pores (pore size of approximately 100–200 μm), while the AM side exhibited a glossy appearance. In addition, due to dislodging of the BAMG, a capsule was formed between the two sides, and the distance between them was approximately 300–500 μm.

### Cell culture, identification, labeling, and immunofluorescence

**Fig. 4:**
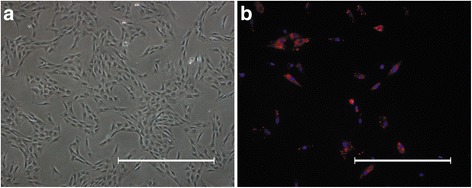
Culture and labelling of ASCs. **a** Photomicrograph of passage 3 ASCs; 40×, *scale bar* = 300 μm (**b**) Photomicrograph of CM-Dil-labelled ASCs; 200×, *scale bar* = 100 μm

After primary ASCs were incubated in culture dishes for 10 days, passage 3 ASCs exhibited a spindle-shaped morphology (Fig. 4a). In our previous study, we showed that the cells isolated from inguinal adipose tissue were ASCs by adipogenic and osteogenic induction [3]. Most of the ASCs were labeled red in the cell membrane and cytoplasm by CM-Dil (Fig. 4b). Immunofluorescence indicated that the ASCs were negative for CK, CD31, SM22α, and NeuN (images not shown).

### Gross evaluation

**Fig. 5:**
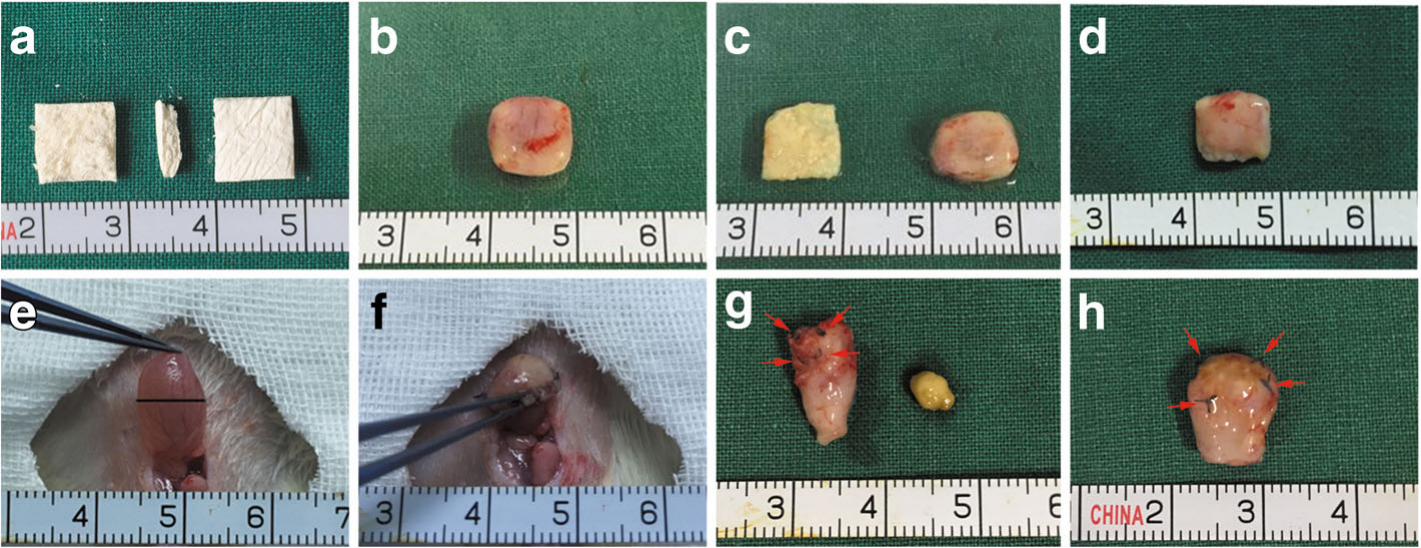
Surgical technique of bladder augmentation with AM-SF-ASC scaffolds and gross morphology of the bladder and urinary stones. **a** Gross view of BAMG-SF prior to implantation. **b** Gross view of BAMG-SF 1 week after subcutaneous prefabrication. **c** Removal of BAMG. **d** Anastomosis of the gap after injecting ASCs into the AM-SF scaffold. **e** 50% supra trigonal cystectomy was performed. **f** Anastomosis of the AM-SF-ASC scaffold into the bladder defect. **g** Gross observation and urinary stones 12 weeks postoperation of the AM-SF group. **h** Gross observation 12 weeks postoperation of the AM-SF-ASC group. The bladder above the black straight line was resected. The *red arrows* denote the sutures between native bladder and the repair area. *Scale bars* = 1 cm

ASCs were seeded to the interior of the AM-SF scaffolds by closing the incision (Fig. 5c and d). Augmentation cystoplasty was a feasible surgical technique for suturing the AM-SF-ASC scaffolds on the bladder defects (Fig. 5e and f). In the control group, obvious contraction (about 50–70%) was found in the regeneration site (Fig. 5 g). In the AM-SF-ASC group, there was slight contraction of the regeneration site 12 weeks after operation (Fig. 5 h). The repair area above is marked by black sutures. We found that the two groups had varying degrees of stone formation (Fig. 5 g), and infrared spectrometer analysis revealed that the calculus composition was ammonium magnesium phosphate (images not shown).

### Cystography

**Fig. 6:**
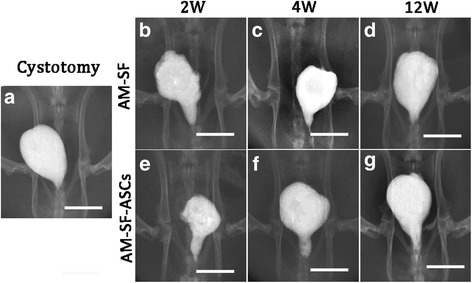
Retrograde cystography of three groups at different time points. Retrograde cystography in **a** the cystotomy group, **b–d** the autologous myofibroblast-silk fibroin (*AM-SF*) group, and **e–g** the AM-SF-adipose-derived stem cell (*AM-SF-ASC*) group at 2 weeks, 4 weeks, and 12 weeks postoperation. *Scale bars* = 1.5 cm

Retrograde cystography in the three groups was performed postoperatively at 2, 4, and 12 weeks (Fig. 6a–g). During the study, no obvious fistulas, diverticulum, or tumors were found in the reconstructed bladders. As time progressed, we found that the bladder wall became smoother and its capacity increased. Furthermore, the shape and capacity of the bladder approached the cystotomy group by 12 weeks after implantation in both control and experimental group (Fig. 6a).

### Histological examination

**Fig. 7:**
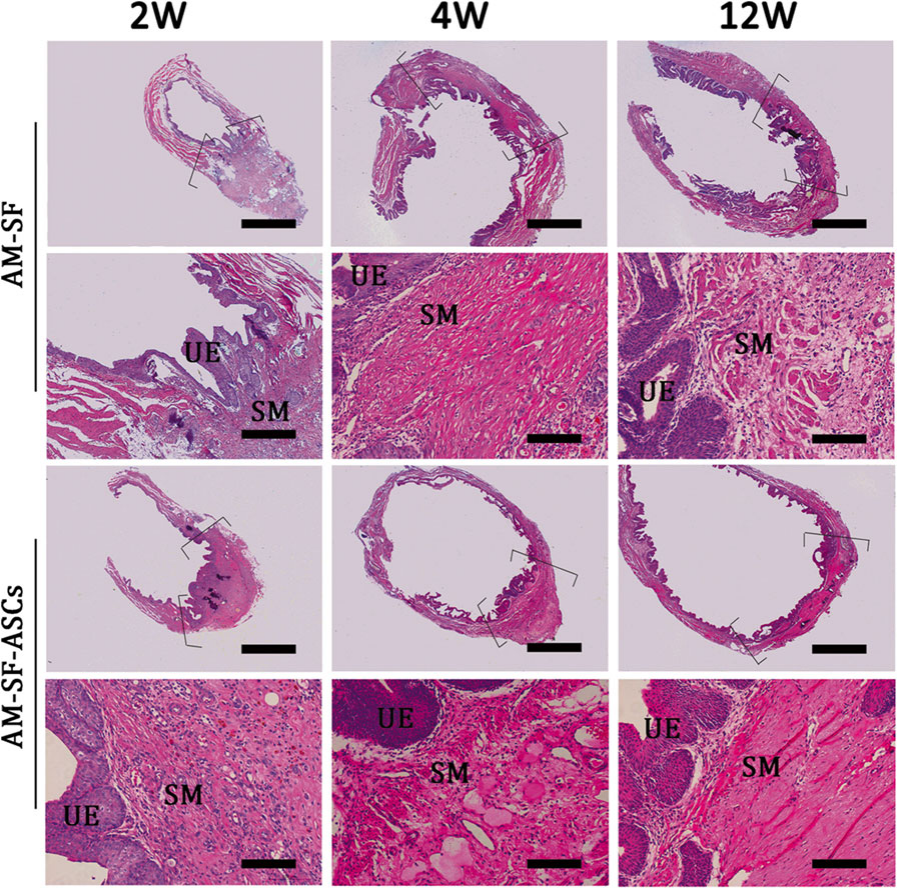
HE analysis of bladder tissue regeneration augmented with autologous myofibroblast-silk fibroin (*AM-SF*) and AM-SF-adipose-derived stem cell (*AM-SF-ASC*) scaffolds 2, 4, and 12 weeks after surgery. *Rows 1 and 3*: Longitudinal photomicrographs of the gross bladder; 25×, *scale bars* = 2 mm. *Brackets* represent sites of original scaffold implantation. *Rows 2 and 4*: Magnification of global tissue regeneration as bracketed in the first and third rows; 200×, *scale bars* = 100 μm. *SM* smooth muscle, *UE* urothelium

The histological examination of bladder tissue sections (HE staining) in the AM-SF and AM-SF-ASC groups from 2 to 12 weeks after implantation illustrated ingrowth of connective tissue into both the marginal and central regions of the original implantation sites (Fig. 7). In addition, the entire urothelium regenerated well in both groups. However, more densely and regularly arranged smooth muscle fibers were detected in the experimental group at 4 and 12 weeks after surgery.

**Fig. 8:**
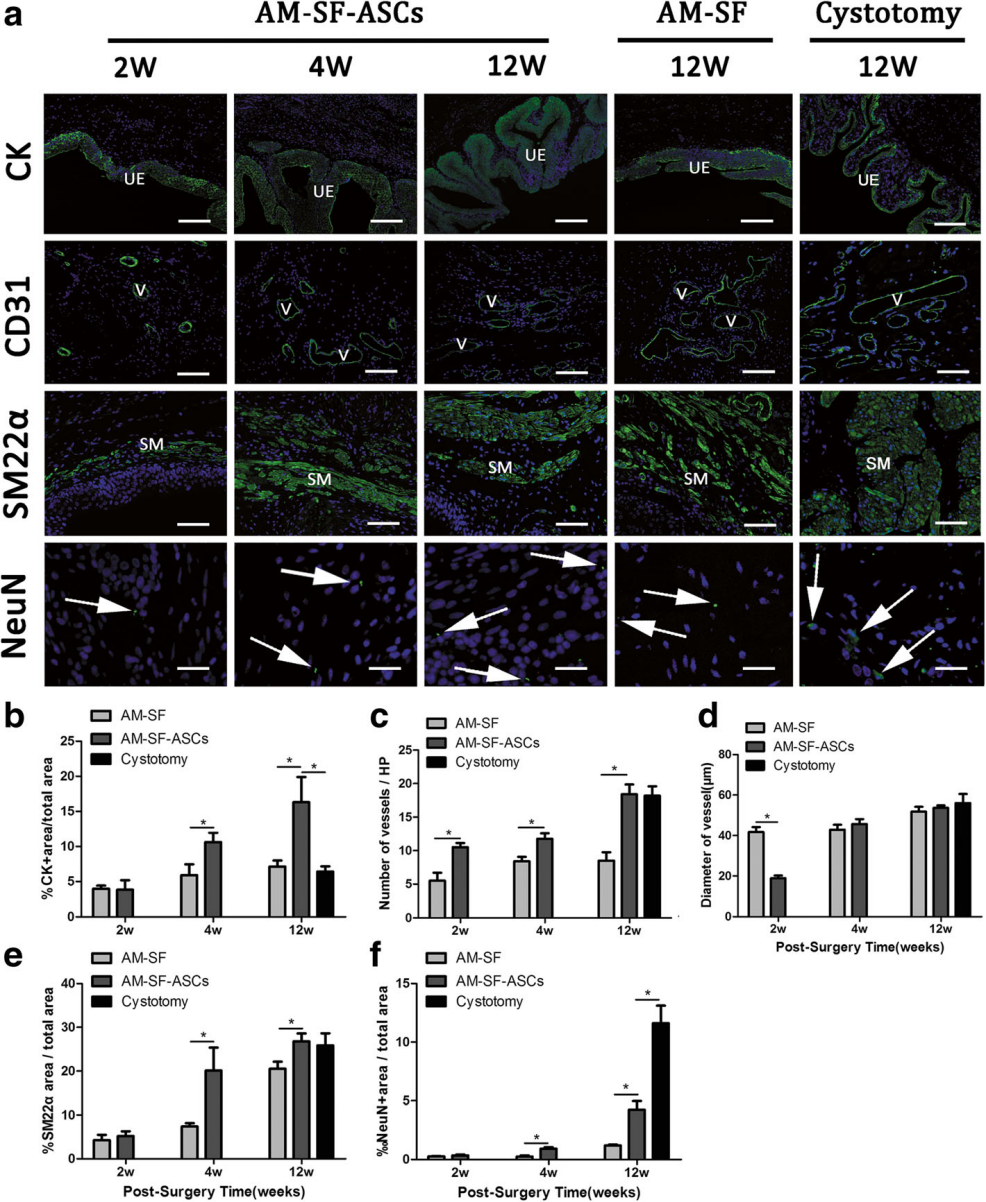
Immunofluorescence assessments of regenerated bladder supported by the autologous myofibroblast-silk fibroin (*AM-SF*) and AM-SF-adipose-derived stem cell (*AM-SF-ASC*) scaffolds and a cystotomy sham. **a** Photomicrographs of cytokeratin (*CK*), a blood vessel endothelial marker (*CD31*), protein expression of smooth muscle 22 alpha (*SM22α*), and a neuronal marker (*NeuN*) in the regenerated bladder tissue. *White arrows* denotes neuronal lineages. For all panels, the respective marker expression is displayed in *green*, and *blue* denotes DAPI nuclear counterstain; 100×, *scale bars* = 200 μm in the CK panels; 200×, *scale bars* = 200 μm in the CD31 and SM22α panels; 400×, *scale bars* = 50 μm in the NeuN panels. Statistical analysis of the extent of regenerated CK+ epithelium (**b**), CD31+ vessels (**c**,**d**), SM22α + smooth muscle bundles (**e**), and NeuN+ neuronal boutons (**f**) present in the original surgical sites of the AM-SF, AM-SF-ASC, and cystotomy sham groups. *p < 0.05 between groups. *SM* smooth muscle, *UE* urothelium, *V* vessels

Immunofluorescence assessments (Fig. 8a) revealed regeneration of the urothelium (CK), smooth muscle bundle (SM22α), vessels (CD31), and nerve bundle (NeuN) at different time points. The percentage of CK+ area/total area at 2 weeks after AM-SF implantation was similar to that in the AM-SF-ASC group (3.97 ± 0.98% versus 3.83 ± 2.33%, *p* > 0.05). The percentages of CK+ area/total area in the control group were significantly less than that in the experiment group after 4 and 12 weeks of regeneration (5.89 ± 3.50% and 7.11 ± 1.76% versus 10.61 ± 2.31% and 16.31 ± 7.14%, respectively, *p* < 0.05; Fig. 8b), and the expression of CK in the AM-SF-ASC group was significantly higher than that in the cystotomy group at 12 weeks (16.31 ± 7.14% versus 6.41 ± 1.47%, *p* < 0.05). In addition, the number of vessels containing CD31-positive endothelial cells in the AM-SF-ASC group improved with time and was significantly higher than that in the AM-SF group at 2, 4, and 12 weeks after implantation (2 weeks: 10.5 ± 1.3/HP versus 5.5 ± 2.4/HP, *p* < 0.05; 4 weeks: 11.8 ± 1.7/HP versus 8.4 ± 1.5/HP, *p* < 0.05; 12 weeks: 18.4 ± 3.3/HP versus 8.5 ± 2.5/HP, *p* < 0.05). No significant difference in the number of vessels between the AM-SF-ASC and cystotomy groups were observed at 12 weeks (18.4 ± 3.3/HP versus 18.2 ± 3.11/HP, respectively, *p* > 0.05; Fig. 8c). The diameter of the vessels increased continuously after the AM-SF and AM-SF-ASC scaffolds were implanted, reaching diameters similar to those in the cystotomy group after 12 weeks (51.78 ± 10.77 μm and 53.62 ± 5.37 μm versus 56.04 ± 10.80 μm, respectively, *p* > 0.05). The diameter of the vessels at 2 weeks in the AM-SF-ASC group was lower than that in the AM-SF group (18.83 ± 6.01 μm versus 41.70 ± 10.97 μm, *p* < 0.05), but at 4 weeks the diameter was increased and was similar to that in the AM-SF group (45.66 ± 10.80 μm versus 42.81 ± 11.11 μm, *p* > 0.05; Fig. 8d).

The regeneration of smooth muscle cells (SMCs) in the marginal region occurred before regeneration in the central zone of the original implantation site. At 2 weeks after implantation, small smooth muscle bundles were observed with a diffused distribution along the edges and near the luminal surface in the marginal region. Histomorphometric analysis (Fig. 8e) revealed that the number of SM22α-positive smooth muscle bundles supported by the AM-SF-ASC scaffolds was similar to that in the AM-SF group (5.17 ± 2.24% versus 4.23 ± 2.76%, *p* > 0.05) at week 2. At 4 weeks after implantation, a denser and more regular arrangement of regenerated smooth muscle bundles invaded the central zone of the original implantation site. The results revealed that the number of de novo SM22α-positive smooth muscle bundles supported by the AM-SF-ASC scaffolds was significantly higher than that supported by the AM-SF scaffolds (20.13 ± 10.43% versus 7.37 ± 1.59%, *p* < 0.05). The number of SM22α-positive smooth muscle bundles continuously increased after implantation in the AM-SF-ASC group, which was still higher at 12 weeks than in the AM-SF group (26.78 ± 3.58% versus 20.52 ± 3.19%, *p* < 0.05) and reached values similar to the cystotomy group (25.86 ± 6.17%, *p* > 0.05; Fig. 8e).

Expression of the neuronal marker NeuN increased with time in both groups (Fig. 8f). Two weeks after implantation, no significant difference was found between the AM-SF-ASC and AM-SF groups (0.35 ± 0.16% versus 0.26 ± 0.08%, *p* > 0.05). However, the number of NeuN-positive cells in the AM-SF-ASC group was significantly higher than that in the AM-SF group at 4 and 12 weeks (4 weeks: 0.92 ± 0.26% versus 0.25 ± 0.17%, *p* < 0.05; 12 weeks: 4.25 ± 1.43% versus 1.19 ± 0.13%, *p* < 0.05), which was still significantly lower than the cystotomy group (11.61 ± 2.99%, *p* < 0.05; Fig. 8f).

### Tracking and differentiation of ASCs

**Fig. 9:**
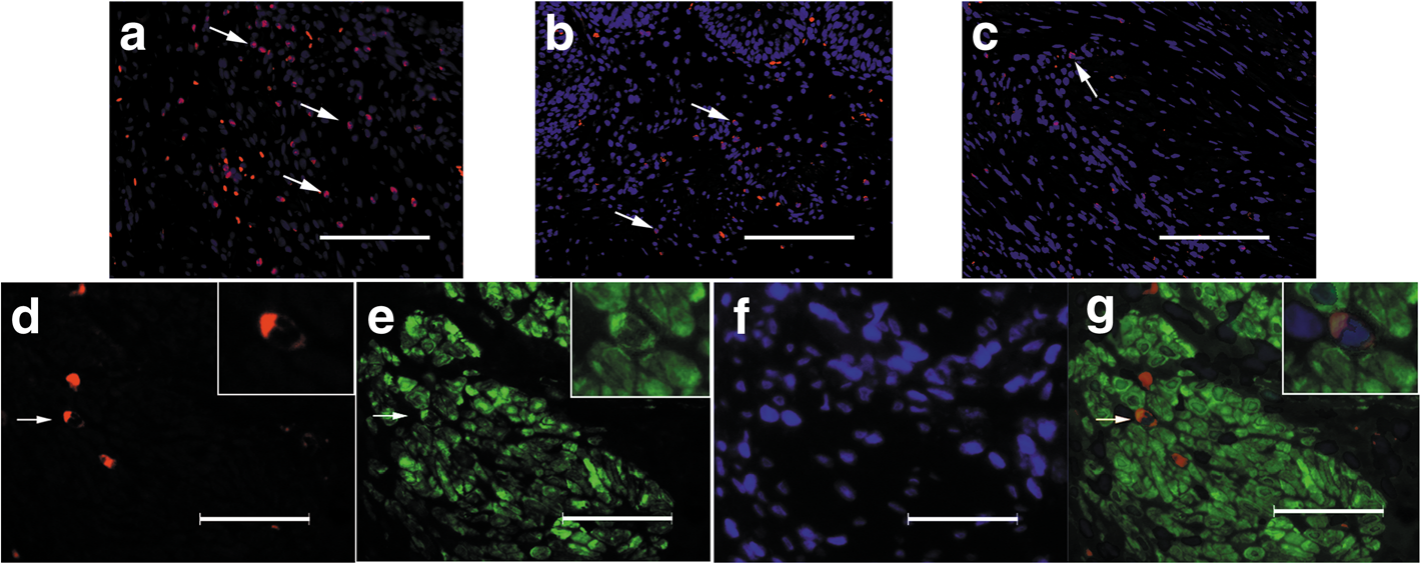
ASC tracking and immunofluorescence of regenerated bladder supported by the AM-SF-ASC scaffolds after surgery. The surviving CM-Dil-labeled ASCs at **a** 2 weeks, **b** 4 weeks, and **c** 12 weeks after surgery; 200×, *scale bar* = 100 μm. **d** Photomicrograph of CM-Dil-labeled ASCs. **e** Photomicrograph of SM22α immunofluorescence. **f** Photomicrograph of nuclei stained with DAPI. **g** Merged images; 400×, *scale bar* = 50 μm. The *white arrows* denote labeled ASCs

The number of surviving labeled ASCs continuously decreased with time after bladder reconstruction with the AM-SF-ASC scaffolds (Fig. 9a–c). Interestingly, a small amount of SM22α + ASCs could be found among smooth muscle bundles (Fig. 9d–g). However, no CD31+/NeuN+/CK+ ASCs were found in our study.

### Bladder function evaluation

**Fig. 10:**
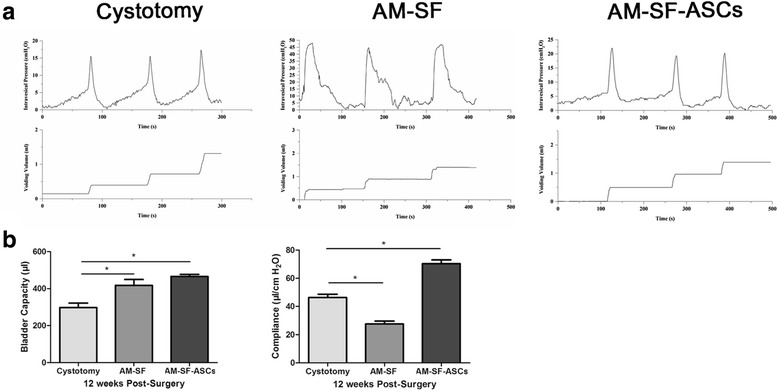
Urodynamics analysis and quantification of urodynamic parameters in augmented bladders 12 weeks after surgery. **a** Representative cystometric tracings of the voiding cycles of the autologous myofibroblast-silk fibroin (*AM-SF*), AM-SF-adipose-derived stem cell (*AM-SF-ASC*), and cystotomy sham group regenerated bladders. **b** Statistical analysis of urodynamic parameters including bladder capacity and compliance. *p < 0.05 between different groups

Urodynamic tracing analysis was performed 12 weeks postoperation in the three groups, and representative cystometric tracings of voiding cycles were found. In the cystotomy and AM-SF-ASC groups, the intravesical pressure gradually increased with the continuous injection of saline, and micturition occurred when the pressure reached a critical value, followed by the V-shaped wave during micturition. The intravesical pressure decreased to baseline at the end of urination (Fig. 10a). However, in the AM-SF group, the intravesical pressure slightly changed with the continuous injection of saline, and no obvious micturition threshold was found. In addition, the intravesical pressure gradually decreased to baseline after the end of urination.

The bladder capacities of the AM-SF and AM-SF-ASCs groups were significantly higher than that of the cystotomy group (418 ± 70 μl and 466 ± 26 μl versus 298 ± 41 μl, respectively, *p* < 0.05), and data showed no significant difference between the AM-SF and AM-SF-ASC groups (*p* > 0.05) (Fig. 10b). The bladder compliance of the AM-SF-ASC group was significantly higher than in the other two groups (70.36 ± 6.21 μl/cmH_2_O versus 27.56 ± 4.69 μl/cmH_2_O and 46.39 ± 4.03 μl/cmH_2_O, respectively, *p* < 0.05).

## Discussion

There are currently two main tissue engineering strategies for bladder reconstruction: one is the use of simple scaffolds, whereas the other is the use of scaffolds seeded with cells or cytokines. Various studies have shown that the latter strategy is far better for restoration of the anatomical structure and function of the bladder [20–22].

However, several issues cannot be solved by the bladder reconstruction method developed in the current study. First, although the bladder tissue regeneration was found using morphology detection, the restoration of bladder function is still difficult. Second, although cell seeding techniques have been applied in many bladder repair experiments, the presence of seeded cells in vivo are scarce after 4 weeks of implantation [3, 23]. Two reasons may account for this phenomenon: either the number of seeded cells is not sufficient, or most of the seeded cells are lost or die due to direct contact with the abdominal cavity or the urine in the bladder lumen.

In this study, the cell-seeding technique was combined with a subcutaneous prefabrication strategy to investigate whether AM-SF scaffolds could promote ASC survival in vivo and restore bladder function. After subcutaneous prefabrication for 1 week, the BAMG-SF was surrounded by transversely arranged myofibroblasts. A bag-shaped AM-SF scaffold structure was acquired by removing the BAMG. This bag-shaped AM-SF scaffold provides sufficient space for ASC implantation, and the dense AM acts as a waterproof barrier that prevents the ASCs from flowing into the abdominal cavity or bladder lumen. Furthermore, the remaining porous SF increases the contact area between the inner wall and the ASCs, which contributes to cells migration and proliferation. We implemented these novel technologies to improve the survival of ASCs and to ensure that ASCs were retained in the repair area. We believe that this novel scaffold may be a promising biomaterial for bladder reconstruction. In addition, the results demonstrated the utility of this technique.

Of the initial 42 rats in this study, four died within the first week after implantation. The survival rate of the two groups was the same, and no rats died in the cystotomy group. Autopsy revealed evidence of dehiscence at the suture line between the scaffold and the native bladder wall and the leakage of urine in the abdominal cavity was obvious. Gross observation of the bladders revealed slight adhesion of the suture area to adjacent fat. Negligible scar formation and graft shrinkage were observed in the regeneration site of the AM-SF-ASCs 12 weeks postoperation. In contrast, marked shrinkage by approximately 50% was observed in the AM-SF group. The contracting of myofibroblasts may account for the shrinkage of the repair area. However, stem cells may prevent myofibroblasts from contracting and prevent tissue fibrosis via a paracrine function [24–26]. In addition, varying degrees of bladder stone formation were observed in the two groups during the second week after implantation. Infrared spectrometer analysis revealed that the calculus composition was ammonium magnesium phosphate, which may be caused by the inflammatory response [27, 28]. No stones were found in the cystotomy group 12 weeks after implantation. Although some studies have suggested that stem cells can attenuate tissue inflammatory responses [29], ASCs were not observed to reduce the rate of stone formation in this study. These findings demonstrated that the inflammation of scaffold materials plays a more important role in stone formation. In addition, degradation of the SF layer was similar to that reported in our previous study [17].

At week 2, there was no significant difference between the two groups regarding regeneration of the urothelium, smooth muscle, or nerve bundles. However, at weeks 4 and 12, the CK/SM22α/NeuN-positive area in the AM-SF-ASC group was superior to that of the control group. Regeneration of the urothelium may be explained as follows: during re-epithelialization, when basal cells undergo initial proliferation and migration across the defect site [5], a small amount of urothelium was found on the luminal surface of the repair area in the two groups at week 2. However, due to the excessive proliferation of basal/intermediate cells [5, 30], a multilayered urothelial lining was detected across the luminal surface in the AM-SF-ASC group at weeks 4 and 12, which may be due to endocrine and paracrine functions of the ASCs. The regeneration of smooth muscle was remarkable in the experimental group, reaching values similar to those of the cystotomy group at week 12. Unfortunately, even after 12 weeks, apparent innervation in the AM-SF-ASC group was still significantly lower than that in the cystotomy group.

Due to the limited transdifferentiation of ASCs, the paracrine and endocrine roles of ASCs at the site of regeneration are more important [31]. Bladder augmentation and construction using the AM-SF-ASCs achieved excellent regeneration of smooth muscle and vessels. However, ASCs possess redundant function on the urothelium and insufficient function on nerves. Nevertheless, the functions of ASCs on neuranagenesis were positive compared to the AM-SF scaffolds. Therefore, ASCs may promote angiogenesis and nerve axon growth in vivo via secreting VEGF, NGF, BDNF, and multiple other cytokines, as previously demonstrated [13].

Urodynamic tracing analysis performed at 12 weeks after implantation demonstrated differences among the three groups. The cystotomy and AM-SF-ASC groups demonstrated more normal waveforms than the control group. Bladder capacity was augmented by least 30% with the AM-SF scaffolds and AM-SF-ASCs. Although nearly half of the bladder was resected, and shrinkage of the regeneration zone was observed in the control group, the bladder itself maintains a certain volume by means of compensation: with decreased strength of the bladder wall, hypertrophy and hyperplasia of the bladder smooth muscle in the normal region may play a major role [32]. Similar to the process of myocardial remodeling, this inevitably results in decreased compliance and ultimately leads to myocardial fibrosis [33]. Hypertrophy and hyperplasia of bladder smooth muscle in the normal region may account for the lower bladder compliance in the AM-SF group, although additional experiments are needed to verify this mechanism. In contrast, in the experimental group, quantification of urodynamic parameters illustrated that bladder capacity and compliance were higher than the cystotomy group, which may be explained by a neurogenic bladder due to limited innervation. The Aδ fibres, which respond to bladder wall distention and trigger micturition [34], were insufficient because of the few innervations in the regeneration site. Thus, the areflexia of de novo regenerated SMCs occurs and eventually leads to a large capacity and low pressure bladder. Therefore, urodynamic normalization of the bladder supported by AM-SF-ASC scaffolds would likely require more than 12 weeks to achieve sufficient innervation.

The highlight of our study is to create a novel AM-SF scaffold consisting of autologous myofibroblasts and porous silk fibroin structure. The scaffolds have the advantage of being water-proof and of a suitable mechanical strength. The bag-shaped structure of the AM-SF scaffold was shown to improve the survival of ASCs in vivo for at least 12 weeks. We confirmed that this novel scaffold combined with ASCs can be successfully used for bladder tissue regeneration and promote the recovery of bladder function. Importantly, ASCs promoted bladder regeneration rapidly and improved urinary function, and possessed the ability to differentiate into mature SMCs in vivo*.* Therefore, we believe that this method has great prospects for future clinical applications.

A limitation of our study is the lack of a comparable result with the simple AM or SF scaffolds. In addition, the technique reported here possesses some drawbacks, such as the complicated procedure for repairing and damage caused by multiple surgeries, the incidence of stone formation, and the limited innervations for bladder regeneration. Future studies will focus on evaluating the long-term efficacy of our procedure in large animal models to translate this scaffold technology to clinical applications.

## Conclusions

This study demonstrated the feasibility of a construct of autologous tissue combined with a porous network biological material and ASCs for use in bladder reconstruction. The bag-shaped structure of the AM-SF scaffold can improve the survival of ASCs to a certain extent. AM-SF scaffolds with ASCs promoted rapid regeneration of the urothelium, smooth muscle, and vessels, and improved bladder compliance and urination function. In addition, the implanted ASCs possessed the ability to differentiate into mature SMCs in vivo. The drawbacks of our study included the high incidence of stone formation and the relatively complicated procedures. Future research should focus on developing a convenient and reliable method in a large animal model with long-term efficacy, and translate this technology to clinical applications.

## Abbreviations

AM: Autologous myofibroblast
ASC: Adipose-derived stem cell
BAMG: Bladder acellular matrix grafts
BDNF: Brain-derived neurotropic factor
CK: Cytokeratin
DMEM: Dulbecco’s modified Eagle’s medium
FBS: Fetal bovine serum
HE: Hematoxylin and eosin
MPO: Myeloperoxidase
NGF: Nerve growth factor
PBS: Phosphate-buffered saline
SD: Sprague-Dawley
SEM: Scanning electron microscopy
SF: Silk fibroin
SMA: Smooth muscle actin
SMC: Smooth muscle cell
VEGF: Vascular endothelial growth factor

## References

[1] Kwon TG, Yoo JJ, Atala A (2008). Local and systemic effects of a tissue engineered neobladder in a canine cystoplasty model. J Urol.

[2] Horst M, Madduri S, Milleret V, Sulser T, Gobet R, Eberli D (2013). A bilayered hybrid microfibrous PLGA—acellular matrix scaffold for hollow organ tissue engineering. Biomaterials.

[3] Zhe Z, Jun D, Yang Z, Mingxi X, Ke Z, Ming Z (2016). Bladder acellular matrix grafts seeded with adipose-derived stem cells and incubated intraperitoneally promote the regeneration of bladder smooth muscle and nerve in a rat model of bladder augmentation. Stem Cells Dev.

[4] Mauney JR, Cannon GM, Lovett ML, Gong EM, Di Vizio D, Gomez P (2011). Evaluation of gel spun silk-based biomaterials in a murine model of bladder augmentation. Biomaterials.

[5] Gomez P, Gil ES, Lovett ML, Rockwood DN, Di Vizio D, Kaplan DL (2011). The effect of manipulation of silk scaffold fabrication parameters on matrix performance in a murine model of bladder augmentation. Biomaterials.

[6] Darby IA, Zakuan N, Billet F, Desmouliere A (2016). The myofibroblast, a key cell in normal and pathological tissue repair. Cell Mol Life Sci.

[7] Bochaton-Piallat ML, Gabbiani G, Hinz B. The myofibroblast in wound healing and fibrosis: answered and unanswered questions. F1000Res. 2016;5. doi:10.12688/f1000research.8190.1. 10.12688/f1000research.8190.1 PMC4847562 27158462

[8] Tomasek JJ, Gabbiani G, Hinz B, Chaponnier C, Brown RA (2002). Myofibroblasts and mechano-regulation of connective tissue remodelling. Nat Rev Mol Cell Biol.

[9] Campbell GR, Turnbull G, Xiang L, Haines M, Armstrong S, Rolfe BE (2008). The peritoneal cavity as a bioreactor for tissue engineering visceral organs: bladder, uterus and vas deferens. J Tissue Eng Regen Med.

[10] Zhang J, Gu GL, Liu GH, Jiang JT, Xia SJ, Sun J (2012). Ureteral reconstruction using autologous tubular grafts for the management of ureteral strictures and defects: an experimental study. Urol Int.

[11] Zuk PA, Zhu M, Mizuno H, Huang J, Futrell JW, Katz AJ (2001). Multilineage cells from human adipose tissue: implications for cell-based therapies. Tissue Eng.

[12] Zhang M, Peng Y, Zhou Z, Zhou J, Wang Z, Lu M (2013). Differentiation of human adipose-derived stem cells co-cultured with urothelium cell line toward a urothelium-like phenotype in a nude murine model. Urology.

[13] Kingham PJ, Kolar MK, Novikova LN, Novikov LN, Wiberg M (2014). Stimulating the neurotrophic and angiogenic properties of human adipose-derived stem cells enhances nerve repair. Stem Cells Dev.

[14] Meng LC, Liao WB, Yang SX, Xiong YH, Song C, Liu LQ (2015). Seeding homologous adipose-derived stem cells and bladder smooth muscle cells into bladder submucosa matrix for reconstructing the ureter in a rabbit model. Transplant Proc.

[15] Hou X, Shi C, Chen W, Chen B, Jia W, Guo Y (2016). Transplantation of human adipose-derived mesenchymal stem cells on a bladder acellular matrix for bladder regeneration in a canine model. Biomed Mater.

[16] Zhang H, Yang R, Wang Z, Lin G, Lue TF, Lin CS (2011). Adipose tissue-derived stem cells secrete CXCL5 cytokine with neurotrophic effects on cavernous nerve regeneration. J Sex Med.

[17] Zhao Y, He Y, Guo JH, Wu JS, Zhou Z, Zhang M (2015). Time-dependent bladder tissue regeneration using bilayer bladder acellular matrix graft-silk fibroin scaffolds in a rat bladder augmentation model. Acta Biomater.

[18] Sharma AK, Bury MI, Fuller NJ, Marks AJ, Kollhoff DM, Rao MV (2013). Cotransplantation with specific populations of spina bifida bone marrow stem/progenitor cells enhances urinary bladder regeneration. Proc Natl Acad Sci U S A.

[19] Tu DD, Seth A, Gil ES, Kaplan DL, Mauney JR, Estrada CR, Jr. Evaluation of biomaterials for bladder augmentation using cystometric analyses in various rodent models. J Vis Exp. 2012:e3981. doi:10.3791/3981. 10.3791/3981 PMC3486757 22907252

[20] Zhang Y, Kropp BP, Lin HK, Cowan R, Cheng EY (2004). Bladder regeneration with cell-seeded small intestinal submucosa. Tissue Eng.

[21] Zhu WD, Xu YM, Feng C, Fu Q, Song LJ, Cui L (2010). Bladder reconstruction with adipose-derived stem cell-seeded bladder acellular matrix grafts improve morphology composition. World J Urol.

[22] Yudintceva NM, Nashchekina YA, Blinova MI, Orlova NV, Muraviov AN, Vinogradova TI (2016). Experimental bladder regeneration using a poly-l-lactide/silk fibroin scaffold seeded with nanoparticle-labeled allogenic bone marrow stromal cells. Int J Nanomedicine.

[23] Talab SS, Kajbafzadeh AM, Elmi A, Tourchi A, Sabetkish S, Sabetkish N (2014). Bladder reconstruction using scaffold-less autologous smooth muscle cell sheet engineering: early histological outcomes for autoaugmentation cystoplasty. BJU Int.

[24] Guo Y, Chen B, Chen LJ, Zhang CF, Xiang C (2016). Current status and future prospects of mesenchymal stem cell therapy for liver fibrosis. J Zhejiang Univ Sci B.

[25] Sangkum P, Yafi FA, Kim H, Bouljihad M, Ranjan M, Datta A (2016). Effect of adipose tissue-derived stem cell injection in a rat model of urethral fibrosis. Can Urol Assoc J.

[26] Matsumoto K, Xavier S, Chen J, Kida Y, Lipphardt M, Ikeda R (2016). Instructive role of the microenvironment in preventing renal fibrosis. Stem Cells Transl Med.

[27] Bury MI, Fuller NJ, Meisner JW, Hofer MD, Webber MJ, Chow LW (2014). The promotion of functional urinary bladder regeneration using anti-inflammatory nanofibers. Biomaterials.

[28] Prywer J, Olszynski M (2016). Bacterially induced formation of infectious urinary stones: recent developments and future challenges. Curr Med Chem.

[29] Bury MI, Fuller NJ, Wethekam L, Sharma AK (2015). Bone marrow derived cells facilitate urinary bladder regeneration by attenuating tissue inflammatory responses. Cent European J Urol.

[30] de Boer WI, Schuller AG, Vermey M, van der Kwast TH (1994). Expression of growth factors and receptors during specific phases in regenerating urothelium after acute injury in vivo. Am J Pathol.

[31] Shukla D, Box GN, Edwards RA, Tyson DR (2008). Bone marrow stem cells for urologic tissue engineering. World J Urol.

[32] Liu C, Xu H, Fu S, Chen Y, Chen Q, Cai Z (2016). Sulforaphane ameliorates bladder dysfunction through activation of the Nrf2-ARE pathway in a rat model of partial bladder outlet obstruction. Oxid Med Cell Longev.

[33] Kemp CD, Conte JV (2012). The pathophysiology of heart failure. Cardiovasc Pathol.

[34] Dorsher PT, McIntosh PM. Neurogenic Bladder. Adv Urol. 2012;2012. doi:10.1155/2012/816274. 10.1155/2012/816274 PMC3287034 22400020

